# Corrigendum: Glacial isostatic uplift of the European Alps

**DOI:** 10.1038/ncomms16138

**Published:** 2017-07-14

**Authors:** Jürgen Mey, Dirk Scherler, Andrew D. Wickert, David L. Egholm, Magdala Tesauro, Taylor F. Schildgen, Manfred R. Strecker

Nature Communications 7: Article number:13382; DOI: 10.1038/ncomms13382 (2016); Published 11 10 2016; Updated 07 14 2017

In an earlier publication, Norton and Hampel proposed post-glacial uplift promoted the re-advance of glaciers at the onset of the Younger Dryas by enlarging their accumulation areas, and estimated a maximum present-day uplift rate due to deglaciation of ∼0.36 mm per year, approximately six times smaller than the value presented in this Article (2.3 mm per year). We suggest this discrepancy is a result of different model assumptions regarding the structure and rheology of the lithosphere, the ice mass and the unloading history.

While this publication was initially omitted from the reference list of this Article, the authors acknowledge that given the differences in the studies' conclusions, citation of this earlier work is wholly appropriate.

Norton, K. P. & Hampel, A. Postglacial rebound promotes glacial re-advances—a case study from the European Alps. *Terra Nova*, **22**, 297–302 (2010).

