# Supplementary material for: Glacial isostatic uplift of the European Alps
Source: Nat Commun. 2016 Nov 10;7:13382. doi: 10.1038/ncomms13382 (PMC5109590; doi:10.1038/ncomms13382)
Supplement: Supplementary Information — Supplementary Figures 1-7, Supplementary Tables 1-2 and Supplementary References. [file ncomms13382-s1.pdf]

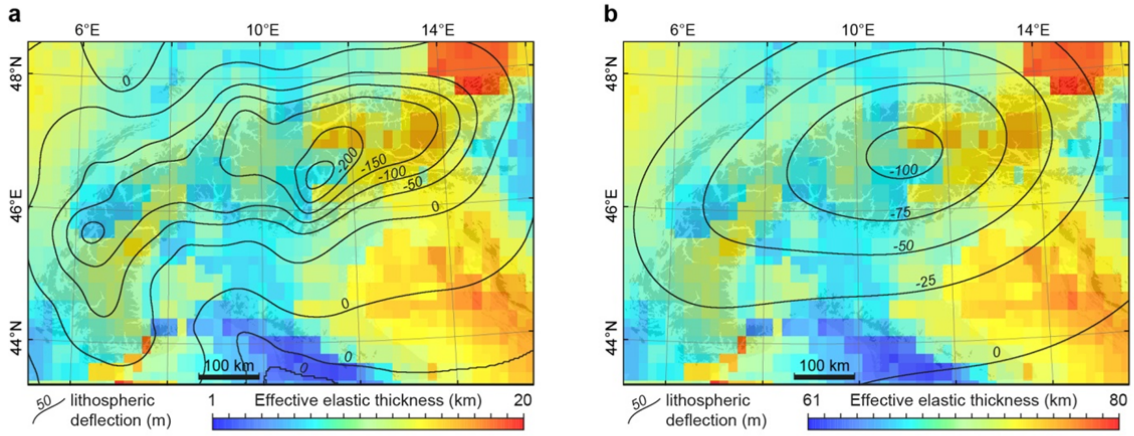


**Supplementary Figure 1 | Deflection of a heterogeneous Alpine lithosphere**. Subsidence due to the load of the LGM icecap for (**a**), a low rigidity[1] (avg. *EET* for the Alps is 10 km) and (**b**), a high rigidity lithosphere (avg. *EET* = 70 km). avg. = average.

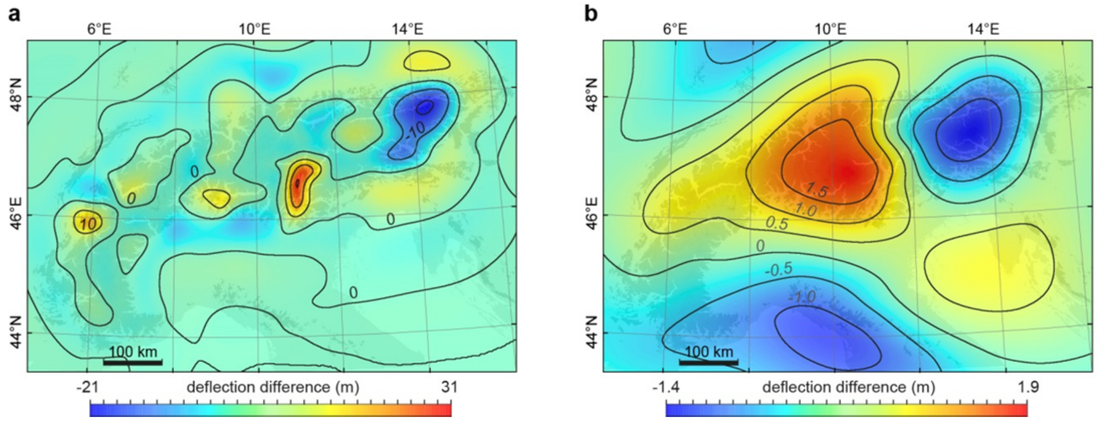


**Supplementary Figure 2 | Effect of spatial variations in *EET***. Difference in lithospheric deflection, relative to a lithosphere with a uniform *EET* of **a,** 10 km and **b,** 70 km.

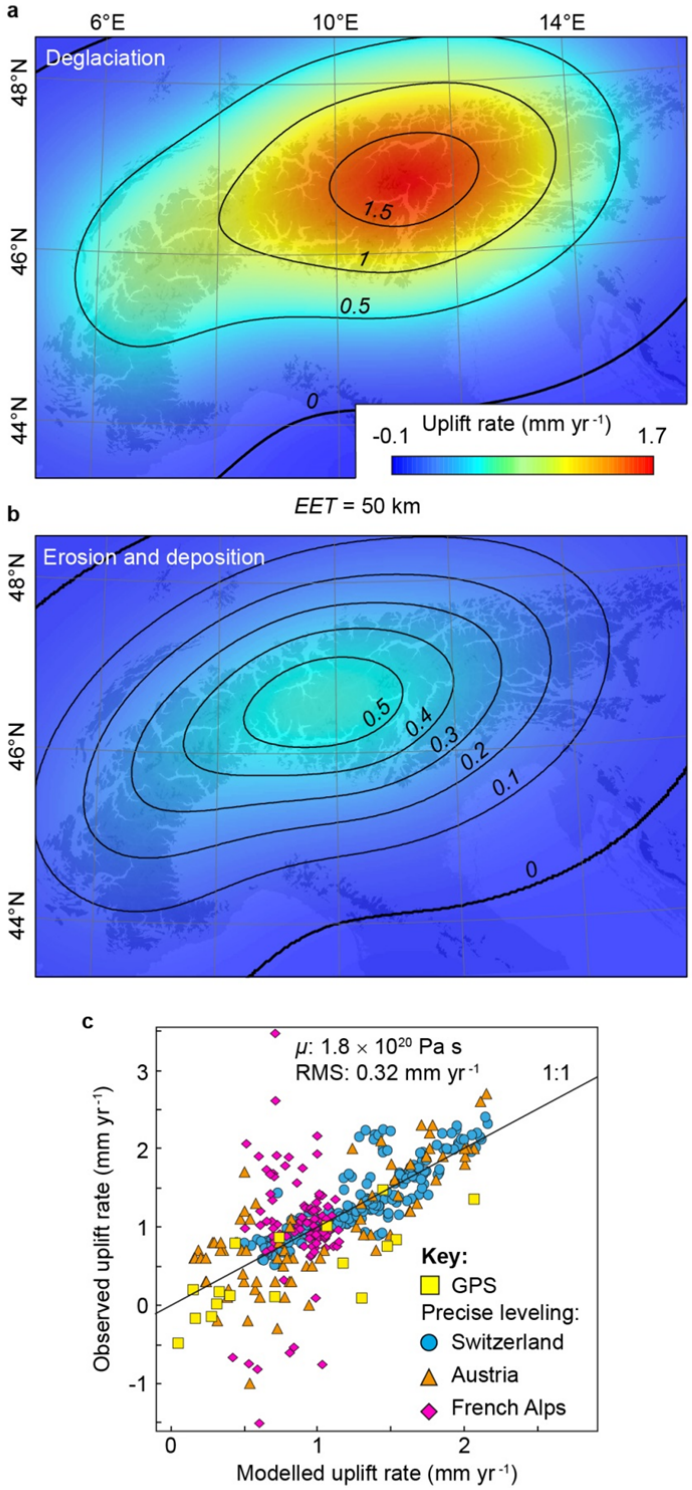


**Supplementary Figure 3 | Results for increased erosional unloading. a**, Deglaciation component. **b**, Uplift rate due to erosional unloading assuming that 90% of the postglacially eroded material has been exported. **c**, Comparison of the combined signal with the geodetic observations[2–5]. Note that the French leveling data was not used in the optimization (see main text for details).

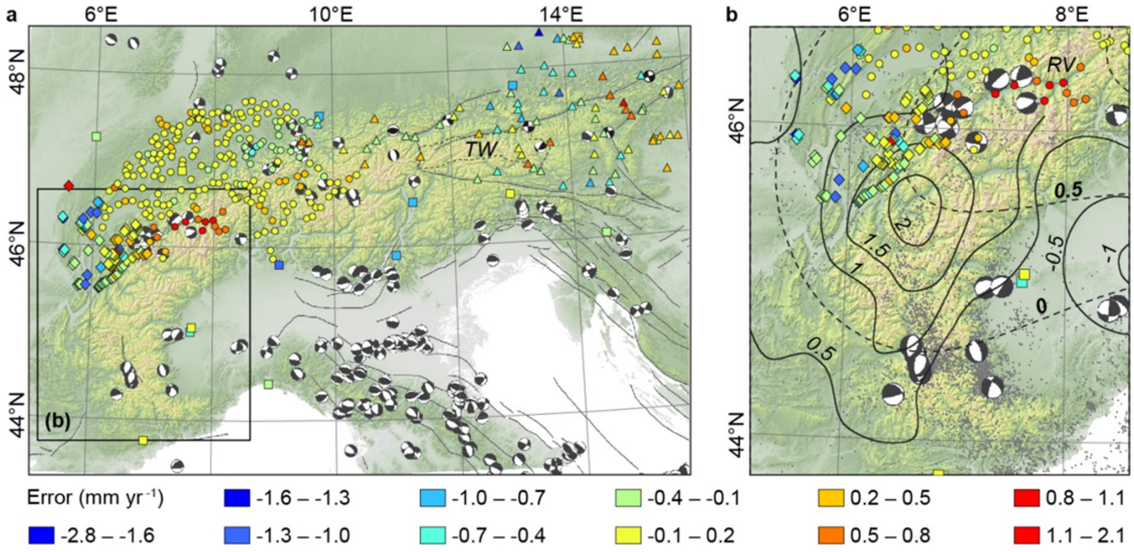


**Supplementary Figure 4 | Model error in a seismotectonic context. a**, Seismogenic faults (black solid lines, http://diss.rm.ingv.it/share-edsf/), and focal plane solutions from the global Centroid-Moment-Tensor catalogue[6] superimposed on a DEM of the study area. Negative errors indicate overestimation. **b**, Inset focusing on the Western Alps. Grey dots: seismicity after NEIC, 1973-2008. Thin solid lines are uplift contours modified after (ref. [7]) and given in mm $yr^{-1}$. Dashed lines show the uplift contours derived in this study (see Fig. 6). RV = Rhône Valley, TW = Tauern Window.

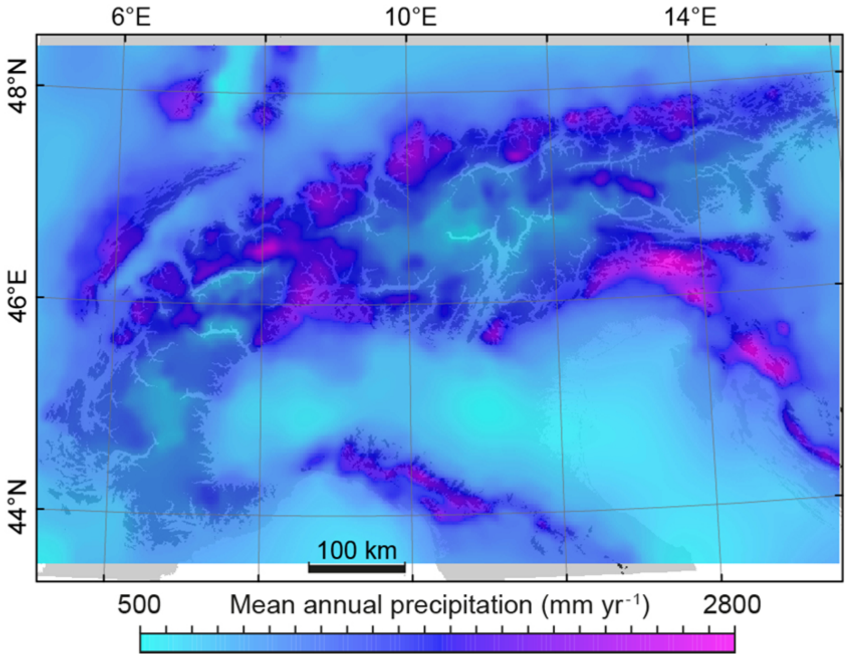


**Supplementary Figure 5 | Map of mean annual precipitation**. Mean annual precipitation was determined from rain-gauge measurements from 1971–2008[8], which were used to impose spatially variable maximum accumulation rates in the ice model.

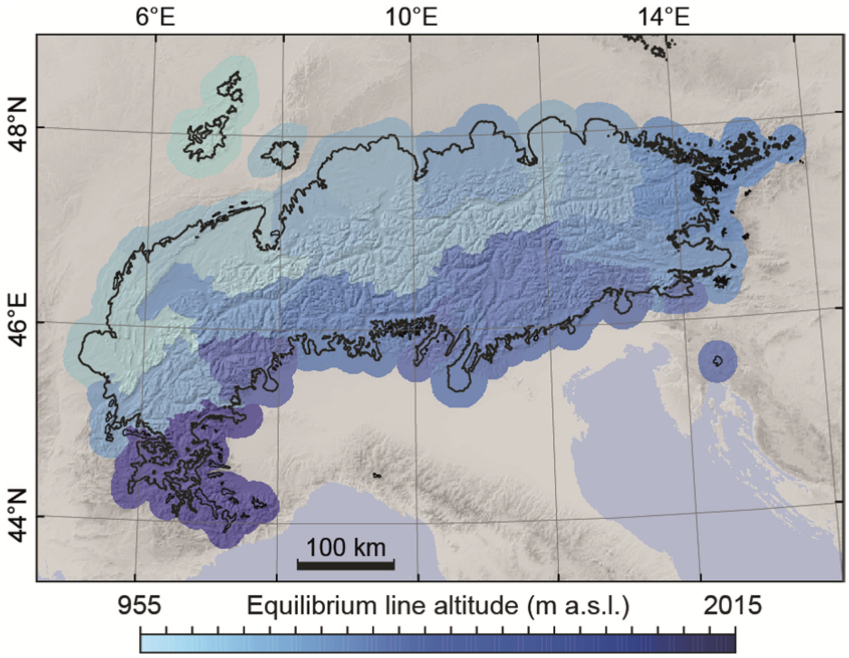


**Supplementary Figure 6 | Map of equilibrium line altitudes.** Distribution of equilibrium line altitudes for the ice model, which best fits LGM ice-geometry indicators, i.e., moraines[9] and trimlines[10–12].

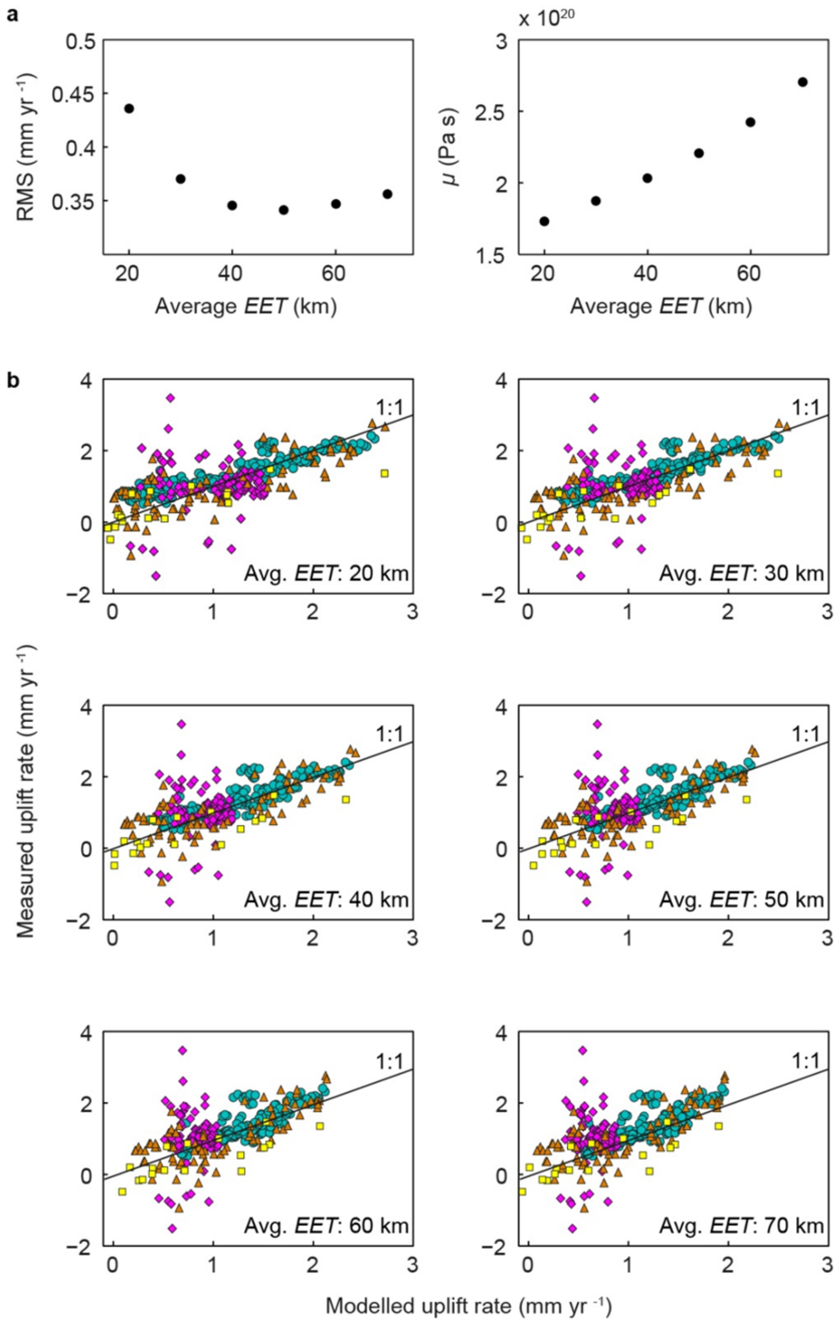


**Supplementary Figure 7 | Sensitivity of viscosity and modelled uplift rates to changes in EET. a,** Root mean squared error (RMS) and viscosity ($\mu$) as a function of the average *EET*. A minimum error occurs at 50 km with $\mu = 2.2 \times 10^{20}$ Pa s. **b,** Modelled versus measured uplift rates for 20, 30, 40, 50, 60, and 70 km of *EET*. Blue circles = Swiss levelling data[2,13]. Orange triangles = Austrian levelling data[3]. Magenta diamonds = French Alps levelling data[5]. Yellow rectangles = permanent GPS data[4]. Avg. = average

**Supplementary Table 1 | Data sources for measurements of valley-fill thicknesses.**

| River catchment | Reference | Method |
|---|---|---|
| Aare | [14] | drilling |
| Adige | [15,16] | drilling, seismic |
| Drau | [17,18] | drilling, seismic |
| Inn | [19–21] | drilling, seismic |
| Isar, Loisach, Lech | [22] | drilling |
| l'Isère | [23] | drilling |
| Reuss, Seez, Linth | [24] | drilling, seismic |
| Rhine | [24–26] | drilling, seismic |
| Rhône | [26,27] | seismic |
| Salzach | [28,29] | drilling, seismic |
| Sarca | [15] | seismic |
| Tagliamento | [30] | drilling, gravimetric |
| Ticino | [26] | seismic |
| Traun, Ens | [29] | drilling |
| Ubaye | [31] | seismic |

**Supplementary Table 2 | Estimates of upper mantle viscosity (*$\mu$*).**

| Region | $\mu$ ($\times 10^{20}$ Pa s) | Reference |
|---|---|---|
| Antarctica | 5 | [32] |
| Hudson Bay | 4 | [33] |
| Fennoscandia | 3–10 | [34] |
| Great Britain | 3–4 | [35] |
| Australian coastline | 2 | [36] |
| European Alps | 1.4–2.8 | this study |
| Basin and Range | 0.18 | [37] |
| Central Andes | 0.01–1 | [38] |
| Japan | 0.5 | [39] |
| Cascadia margin | 0.05–0.5 | [40] |
| Iceland | 0.01–0.5 | [41] |

## Supplementary References


1. Tesauro, M., Kaban, M. K. & Cloetingh, S. A. P. L. How rigid is Europe's lithosphere? *Geophys. Res. Lett.* **36,** 1–6 (2009).

2. Schlatter, A., Schneider, D., Geiger, A. & Kahle, H. G. Recent vertical movements from precise levelling in the vicinity of the city of Basel, Switzerland. *Int. J. Earth Sci.* **94,** 507–514 (2005).

3. Ruess, D. & Mitterschiffthaler, P. Rezente Höhenänderungen in Österreich abgeleitet aus geodätischen Wiederholungsmessungen. in *18. Internationale Geodätische Woche, Obergurgl* (eds. Hanke, K. & Weinhold, T.) 111–123 (Wichmann, 2015).

4. Bruyninx, C. The EUREF Permanent Network: a multi-disciplinary network serving surveyors as well as scientists. *GeoInformatics* **7,** 32–35 (2004).

5. Jouanne, F., Ménard, G. & Darmendrail, X. Present-day vertical displacements in the north-western Alps and southern Jura Mountains: Data from leveling comparisons. *Tectonics* **14,** 606–616 (1995).

6. Ekström, G., Nettles, M. & Dziewoński, A. M. The global CMT project 2004–2010: Centroid-moment tensors for 13,017 earthquakes. *Phys. Earth Planet. Inter.* **200–201,** 1–9 (2012).

7. Nocque Jt, . *et al.* Present-day uplift of the western Alps. *Nat. Sci. Rep.* (2016).

8. Isotta F, . A. *et al.* The climate of daily precipitation in the Alps: development and analysis of a high-resolution grid dataset from pan-Alpine rain-gauge data. *Int. J. Climatol.* **34,** 1657–1675 (2014).

9. Ehlers, J. & Gibbard, P. L. *Quaternary glaciations— extent and chronology, Part I Europe*. (Elsevier, 2004).

10. Kelly, M. A., Buoncristiani, J. F. & Schlüchter, C. A reconstruction of the last glacial maximum (LGM) ice-surface geometry in the western Swiss Alps and contiguous Alpine regions in Italy and France. *Eclogae Geol. Helv.* **97,** 57–75 (2004).

11. Florineth, D. Surface geometry of the Last Glacial Maximum (LGM) in the southeastern Swiss Alps (Graubünden) and its paleoclimatological significance. *Eiszeitalter und Gegenwart* **48,** 23–27 (1998).

12. Florineth, D. & Schlüchter, C. Reconstructing the Last Glacial Maximum (LGM) ice surface geometry and flowlines in the Central Swiss Alps. *Eclogae Geol. Helv.* **91,** 391–407 (1998).

13. Kahle, H.-G. *et al.* in *NRP 20—Deep Structure of the Swiss Alps* (eds. Pfiffner, O. A., Lehner, P., Heitzmann, P., Mueller, S. & Steck, A.) 251–259 (Birkhäuser, 1997).

14. GEOSOND. GEOSOND: Geologische Sondierungen im Kanton Bern. (2014). at <http://www.be.ch/geoportal>

15. Faccioli, E. & Vanini, M. Complex seismic site effects in sediment-filled valleys and implications on design spectra. *Prog. Struct. Eng. Mater.* **5,** 223–238 (2003).

16. Bassetti, M. & Borsato, A. Evoluzione geomorfologica della Bassa Valle dell' Adige dall' Ultimo Massimo Glaciale: sintesi delle conoscenze e riferimenti ad aree limitrofe. *Stud. trentini di Sci. Nat. - Acta Geol.* **82,** 31–42 (2005).

17. Brückl, E., Brückl, J., Chwatal, W. & Ullrich, C. Deep alpine valleys: Examples of geophysical explorations in Austria. *Swiss J. Geosci.* **103,** 329–344 (2010).

18. Heinz, H. & Walach, G. Ergebnisse refraktionsseismischer Messungen im Gebiet des Lurnfeldes (Drautal, Oberkärnten). *Verh. Geol. B.-A.* **2,** 77–83 (1979).

19. Aric, K. & Steinhauser, P. Geophysikalische Untersuchung des Inntal-Untergrundes bei Thaur, östlich von Innsbruck. *Zeitschrift für Gletscherkd. und Glazialgeol.* **12,** 37–54 (1976).

20. Preusser, F., Reitner, J. M. & Schlüchter, C. Distribution, geometry, age and origin of overdeepened valleys and basins in the Alps and their foreland. *Swiss J. Geosci.* **103,** 407–426 (2010).

21. Steinbrener, J. Sedimentologische und geochemische Untersuchung der Tiefbohrung Wattens I (Tirol). (Universität Wien, 2011).

22. Frank, H. Glazial übertiefte Täler im Bereich des Isar-Loisach-Gletschers. *Eiszeitalter und Gegenwart* **29,** 77–99 (1979).

23. Nicoud, G., Royer, G., Corbin, J., Lemeille, F. & Paillet, A. Creusement et remplissage de la vallée de l'Isère au Quaternaire récent. Apports nouveaux du forage GMB1 (1999) dans la région de Grenoble (France). *Géologie la Fr.* **4,** 39–49 (2002).

24. Wildi, W. Isohypsenkarte der quartären Felstäler in der Nord- und Ostschweiz mit kurzen Erläuterungen. *Eclogae Geol. Helv.* **77,** 541–551 (1984).

25. Eberle, M. Zur Lockergesteinsfüllung des St . Galler und Liechtensteiner Rheintales. *Eclogae Geol. Helv.* **80,** 193–206 (1987).

26. Pfiffner, O. A. *et al.* in *Deep Structure of the Swiss Alps: results of NRP 20* (eds. Pfiffner, O. A., Heitzmann, P., Lehner, P., Mueller, S. & Steck, A.) 265–288 (Birkhäuser, 1997).

27. Finckh, A. & Frei, P. Seismic reflection profiling in the Swiss Rhone valley. Part 1, Seismic reflection field work, seismic processing and seismic results of the Roche-Vouvry and Turtmann and Agarn lines. *Eclogae Geol. Helv.* **84,** 345–357 (1991).

28. Bleibinhaus, F., Hilberg, S. & Stiller, M. First results from a seismic survey in the Upper Salzach Valley, Austria. *Austrian J. Earth Sci.* **103,** 28–32 (2010).

29. van Husen, D. Verbreitung, Ursachen und Füllung glazial übertiefter Talabschnitte an Beispielen in den Ostalpen. *Eiszeitalter und Gegenwart* **29,** 9–22 (1979).

30. Barnaba, C. *et al.* The buried shape of an alpine valley from gravity surveys, seismic and ambient noise analysis. *Geophys. J. Int.* **180,** 715–733 (2010).

31. Jongmans, D. & Campillo, M. The response of the Ubaye Valley (France) for incident SH and SV waves: Comparison between measurements and modeling. *Bull. Seismol. Soc. Am.* **83,** 907–924 (1993).

32. Argus, D. F., Peltier, W. R., Drummond, R. & Moore, A. W. The Antarctica component of postglacial rebound model ICE-6G_C (VM5a) based on GPS positioning, exposure age dating of ice thicknesses, and relative sea level histories. *Geophys. J. Int.* **198,** 537–563 (2014).

33. Mitrovica, J. X. & Forte, A. M. A new inference of mantle viscosity based upon joint inversion of convection and glacial isostatic adjustment data. *Earth Planet. Sci. Lett.* **225,** 177–189 (2004).

34. Nordman, M., Milne, G. & Tarasov, L. Reappraisal of the Angerman River decay time estimate and its application to determine uncertainty in Earth viscosity structure. *Geophys. J. Int.* **201,** 811–822 (2015).

35. Lambeck, K., Smither, C. & Johnston, P. Sea-level change, glacial rebound and mantle viscosity for northern Europe. *Geophys. J. Int.* **134,** 102–144 (1998).

36. Nakada, M. & Lambeck, K. Late Pleistocene and Holocene sea-level change in the Australian region and mantle rheology. *Geophys. J. R. Astron. Soc.* **96,** 497–517 (1989).

37. Bills, B. G., Currey, D. R. & Marshall, G. A. Viscosity estimates for the crust and upper mantle from patterns of lacustrine shoreline deformation in the Eastern Great Basin. *J. Geophys. Res.* **99,** 22059–22086 (1994).

38. Bills, B. G. *et al.* Hydro-isostatic deflection and tectonic tilting in the central Andes: Initial results of a GPS survey of Lake Minchin shorelines. *Geophys. Res. Lett.* **21,** 293–296 (1994).

39. Okuno, J. & Nakada, M. in *Dynamics of the Ice Age Earth: A Modern Perspective* (ed. Wu, P.) 443–458 (Trans Tech Publications, 1998).

40. James, T. S., Clague, J. J., Wang, K. & Hutchinson, I. Postglacial rebound at the northern Cascadia subduction zone. *Quat. Sci. Rev.* **19,** 1527–1541 (2000).

41. Sigmundsson, F. & Einarsson, P. Glacio-isostatic crustal movements caused by historical volume change of the Vatnajökull Ice Cap, Iceland. *Geophys. Res. Lett.* **19,** 2123–2126 (1992).
